# MiR-214 increases the sensitivity of breast cancer cells to tamoxifen and fulvestrant through inhibition of autophagy

**DOI:** 10.1186/s12943-015-0480-4

**Published:** 2015-12-15

**Authors:** Xinfeng Yu, Aiping Luo, Yicong Liu, Shuqing Wang, Ye Li, Wenna Shi, Zhihua Liu, Xianjun Qu

**Affiliations:** Department of Pharmacology, School of Basic Medical Sciences, Capital Medical University, No.10, Xitoutiao, Youanmenwai Avenue, 100069 Beijing, China; State Key Lab of Molecular Oncology, Cancer Institute & Hospital, Chinese Academy of Medical Sciences, Beijing, China; Department of Pharmacology, School of Chemical Biology & Pharmaceutical Sciences, Capital Medical University, Beijing, China

**Keywords:** ER^+^ breast cancer, Endocrine resistance, Apoptosis, Autophagy, MiR-214, UCP2

## Abstract

**Background:**

Tamoxifen (TAM) and fulvestrant (FUL) are the major drugs for patients with estrogen receptor-positive (ER^+^) breast cancers. However, the development of endocrine resistance is the impediment for successful treatment. We aimed to explore the mechanisms of endocrine resistance and therapeutic strategy for overcoming resistance against TAM and FUL.

**Methods:**

Experiments were performed in ER^+^ and estrogen/TAM-sensitive MCF7 cells and antiestrogen-resistant MCF7/LCC9 cells. The expression of miR-214 and uncoupling protein 2 (UCP2) was determined by RT-qPCR and Western blot in breast cancer cells and human breast cancer tissue specimens. Cell autophagy was examined by fluorescent probe monodansyl cadaverine (MDC) and GFP-LC3-II-positive punctate identified by confocal microscopy. Apoptotic cells were determined by Annexin V-FITC/PI staining. The potential regulatory target of miR-214 was determined by prediction tool, target protein expression and luciferase reporter assay.

**Results:**

4-OHT/FUL treatment resulted in induction of apoptosis as well as autophagy in breast cancer cells. Autophagy might be the major cause of endocrine resistance to 4-OHT or FUL. MiR-214 increased the sensitivity of breast cancer cells to the 4-OHT/FUL-induced apoptosis through inhibition of autophagy. Importantly, a negative correlation was established between miR-214 and UCP2 in human breast cancer tissue specimens assayed by RT-qPCR. UCP2 was identified to be a direct target of miR-214. Further study in MCF7/LCC9 cells indicated that endocrine resistance might arise from activation of the PI3K-Akt-mTOR pathway, thereby inducing autophagy by overexpression of UCP2.

**Conclusion:**

MiR-214 increased the sensitivity of breast cancer cells to TAM and FUL through inhibition of autophagy by targeting UCP2. MiR-214 shows potential as a novel therapeutic strategy for overcoming endocrine resistance in ER^+^ breast cancers.

## Background

Breast cancer is one of the most prevalent cancers in women worldwide, with almost 1.2 million new cases diagnosed each year [1]. Breast cancers are classified based on the expression of hormone receptors: estrogen receptor (ER), progesterone receptor (PR), and human epidermal growth receptor 2 (HER2). Among those patients, about 70 % of cases express ER. In patients with metastatic breast cancer, the percentage of cases with ER reaches 75 % [2]. Targeting estrogen and its receptors is therefore an important strategy in endocrine therapies for breast cancers. Commonly used drugs include (a) tamoxifen (TAM) and (b) fulvestrant (FUL). TAM has been used as an anti-estrogen agent for many years. TAM physically competes with estrogens to bind to ER, thereby preventing the proliferative stimuli of estrogens [3]. Unlike TAM, FUL is a pure ER antagonist which competitively binds to ER, leading to its downregulation in cancer cells. FUL is specifically indicated for treatment of postmenopausal patients with ER^+^ metastatic breast cancer [4, 5].

Although these drugs are initially effective, overall clinical benefit from use of them is often eventually limited by the development of endocrine resistance. TAM, for instance, is not effective in approximately 30 % of patients and resistance is observed in 50 % of patients eventually after treatment [6]. After prolonged therapy with FUL, therapy resistance eventually develops in most of patients and recurs with metastatic disease [5, 7]. Multiple mechanisms responsible for endocrine resistance are recognized, such as deregulation of the ER pathway itself, alterations in cell cycle and survival molecules, and altered expression of miRNAs [8, 9]. Recently, the noteworthy mechanism of endocrine resistance is the increase of autophagy and apoptosis in the ER^+^ breast cancers following TAM treatment [10]. In fact, autophagy and apoptosis pathways are tightly connected with each other by substantial interplays, enabling the coordinated regulation of cell fates by these two pathways. Studies have shown that autophagy occurs when the apoptotic machinery is imperfect, detailed mechanisms underlying are not clearly understood [11, 12]. Autophagy is an evolutionarily conserved mechanism of cellular self-digestion in which proteins and organelles are degraded through delivery to lysosomes. The process of autophagy pathway is regulated by a variety of signaling molecules [13]. Since autophagy is involved in the process of pathological disorders with a shift in balance between cell death and survival in response to TAM, increase of autophagy was thus recognized as an important mechanism of TAM resistance [14]. Inhibition of autophagy might potentiate resensitization of previously antiestrogen resistant breast cancer cells [6]. However, a balance might exist between autophagy and apoptosis in response to the endocrine therapy. The key point in the determination of cell fate appears to be affected by the status of cancer cells in autophagy or apoptosis [15].

MiRNAs are a class of short, endogenous, noncoding RNAs (~20–24 nucleotides) that regulate the expression of a wide variety of genes. Through base pairing with the 3′-untranslated region (3′UTR) of target genes, miRNAs were found to enhance mRNA degradation or inhibit posttranscriptional translation [16, 17]. Mountains of reports showed that miRNA alteration or dysfunction might play important roles in tumorigenesis and cancer metastasis by the way of regulating cancer cell proliferation, differentiation, apoptosis and invasion [18]. Notably, miRNAs were found to function in the regulation of autophagy. For instance, miR-101 was identified as a potent inhibitor of basal, etoposide- and rapamycin-induced autophagy [19]. MiR-214 is often dysregulated in various cancers and its functions vary largely with tissue types. The upregulation of endogenous miR-214 has been linked with increased proliferation, migration, invasion, extravasation and metastasis in specific cancer types, such as pancreatic, cervical, hepatocellular, gastric, prostate and ovarian cancer [20]. By contrast, the downregulation of this miR has been associated with cancer progression and metastasis in some other caner types, such as breast cancers, suggesting that tight regulation of miR-214 is important for cellular function [21, 22]. In addition, miR-214 has been shown to induce apoptosis in some cancer types [23]. In our pilot study, miR-214 was found downregulated in human breast cancer tissue specimens. Using ER^+^ breast cancer cell lines, miR-214 increased the sensitivity of cancer cells to TAM and FUL through inhibition of autophagy. Upregulation of miR-214 led to the inhibition of breast cancer growth and invasion [22]. MiR-214 was thus considered as a tumor suppressor in many cancers. However, functional relevance of these findings and mechanisms has not been fully addressed. In this study, we examined the effects of miR-214 on sensitivity of breast cancer cells to TAM and FUL in the ER^+^ and estrogen/TAM-sensitive MCF7 cells and antiestrogen-resistant MCF7/LCC9 cells. The biochemical mechanisms behind the effects of miR-214 were then investigated.

## Methods

### Human breast cancer tissue specimens

This section of experiment was approved by the Institutional Review Board of the Chinese Academy of Medical Sciences Cancer Institute, and written informed consents were obtained from all patients. No patients received any type of endocrine therapy. Twenty pairs of breast cancer tissue specimens and adjacent normal mucosa were obtained from Chinese Academy of Medical Sciences Cancer Hospital (Beijing, China). Each pair of cancer specimen and adjacent normal tissue was confirmed by pathological analysis. All tissues were immediately frozen in liquid nitrogen after surgical removal until use for real-time-qPCR analysis.

### Cell culture and drug treatment

Human breast cancer cell line MCF7 was purchased from American Type Culture Collection (ATCC, Rockville, MD). MCF7 is the ER positive and estrogen/TAM-sensitive cell line. MCF7 cells were grown in DMEM medium supplemented with 10 % fetal bovine serum (FBS) and 0.01 mg/ml insulin at 37 °C in a humid atmosphere (5 % CO_2_-95 % air). MCF7/LCC9 cell line is a fully antiestrogen-resistant MCF7 variant exhibiting cross resistance to FUL and TAM [24]. MCF7/LCC9 was provided by Dr. Robert Clarke (Georgetown University). MCF7/LCC9 cells were maintained in phenol-red free DMEM containing 5 % dextran charcoal-stripped FBS (DCC-FBS) and 100 μg/ml penicillin.

Prior to treatment, cells were exposed to 5 % DCC-FBS for 48 h (serum-starved). Cells were then treated with 5 μΜ 4-hydroxytamoxifen (4-OHT, Sigma-Aldrich) or 1 μM FUL (Sigma-Aldrich) for 48 or 72 h in the presence or absence of 5 mM 3-methyladenine (3-MA, Sigma-Aldrich). 0.1 % ethanol was used as vehicle control (0.01 % final volume).

### Plasmids construction and cell transfection

To produce UCP2 expression plasmid, full length of UCP2 coding region was amplified by PCR using sequence-specific primers (forward: 5’-cgcGGATCCaggacgtagcaggaaatcagc-3’, reverse: 5’-gcTCTAGAagaggtgatcaggtcagcag-3’, containing restriction sites for BamH1 and XbaI respectively). The double digested cDNA was inserted into pcDNA3.1-HisC plasmid and confirmed by sequencing. For UCP2 knockdown assay, the siRNA against UCP2 was synthesized by GenePharma (Shanghai, China). The oligonucleotide sequences were 5’-GCACCGUCAAUGCCUACAATT-3’. MiR-214 mimics and inhibitors were also purchased from GenePharma (Shanghai, China). Transient transfection of UCP2 plasmid and siRNA or miRNA was performed with Lipofectamine 2000 (Invitrogen) according to manufacturer’s instructions. MCF7 or MCF7/LCC9 cells were transfected either with 200 nM UCP2 siRNA or 100 nM miR-214 mimics/inhibitors for 48 h. Corresponding negative control was concurrently used in all experimental system. Cells were harvested for RT-PCR or Western blotting analysis.

### RNA extraction and RT-qPCR

Total RNA was extracted using TRIzol (Invitrogen) in accordance with manufacturer’s protocol. Reverse transcription of total miRNA was performed by using a miScript reverse transcription kit (Qiagen). MiScript SYBR Green PCR kit (Qiagen) together with a pair of miR-214 specific primers was used for mature miRNA detection. RNU6B was used as an internal normalized reference.

For detection of UCP2 mRNA expression, total RNA was reversely transcribed by using SuperScript III First-Strand Synthesis System (Invitrogen) according to manufacturer’s instruction. Real-time PCR was performed in triplicate in an ABI 7900HT real-time PCR system (Applied Biosystems) with a 20 μl reaction mixture containing Brilliant II SYBR Green qPCR master mix and 300 nM UCP2 primers. Primer sequences for UCP2 were 5’-cgcatcggcctgtatgattc-3’ and 5’- cataggtcaccagctcagc-3’. Primer sequences for GAPDH were 5’-gagtcaacggatttggtcgt-3’ and 5’-ttgattttggagggatctcg-3’. The thermal cycling was initiated by polymerase activation step for 10 min at 95 °C followed by 40 cycles of denaturation (95 °C for 30 s) and annealing/extension (60 °C for 1 min). Relative expression of UCP2 was normalized to GAPDH as an internal control and determined by a previously described method [25].

### Western blotting analysis

Cells were harvested and lysed in radioimmune precipitation assay (RIPA) buffer. Protein was extracted and concentration was determined by using a BCA kit (Thermo Fisher Scientific). Equal amounts of cell lysates were resolved by SDS-PAGE and transferred to PVDF membranes (Millipore). After blocking in 5 % nonfat milk for 1 h, the membranes were immunoblotted overnight at 4 °C with primary antibodies against LC3, beclin-1, caspase-9, Bcl-2, PARP (Cell Signaling Technology) and Bax (Santa Cruz) followed by incubation with polyclonal HRP-conjugated secondary antibodies for 1 h at room temperature. Immunoreactive products were visualized by chemiluminescence (Pierce Biotechnology) and quantified by densitometry using Quantity One software. Densitometric analyses of bands were normalized with β-actin functioning as a loading control.

### Cell viability assay

MCF7 cells seeded in 6-well plate were transfected with 100 nM negative control or miR-214 mimics and inhibitors for 24 h. Cells were trypsinized into 96-well plates at a density of 8 × 10^3^ cells/well and then treated with 5 μΜ 4-OHT, or 1 μM FUL for 72 h.. Cell viability was estimated by the 3-[4,5-dimethylthiazol-2-yl]-2,5-diphenyltetrazolium bromide (MTT) assay.

### Annexin V-FITC/PI staining assay

MCF7 cells grown in 6-well plate were transfected with 2 μg UCP2 expression plasmid, 200 nM UCP2 siRNA or 100 nM miR-214 mimics and inhibitors as well as corresponding negative control for 24 h. Cells were then treated with 5 μM 4-OHT for additional 48 h in 5 % DCC-FBS/DMEM. Cells were harvested and washed with cold PBS. Levels of phosphatidylserine on cell surface were quantitatively estimated by using Annexin V-fluorescein isothiocyanate (FITC) and Propidium Iodide (PI) apoptosis detection kit according to manufacturer’s instruction (Bioscience). Apoptotic cells were analyzed in a flow cytometer (Guava easyCyte) using FL1 (excitation 488 nm, green) and FL3 (excitation 585 nm, red) channels. A minimum of 10,000 events was collected for each sample. Results were expressed as percentage of apoptotic cells relative to total cells.

### Cell autophagy analysis

Cells were transfected with GFP-LC3 plasmid (Addgene) and then treated with 0.1 % v/v ethanol vehicle or 5 μM 4-OHT or 1 μM FUL for 48 h. GFP-LC3-II-positive punctate pattern was observed under confocal microscope (Leica TCS SP5) equipped with oil immersion lens (40×) with 405- and 488-nm excitation lasers. Numbers of autophagosomes were counted by using the Image J program (National Institutes of Health).

### Staining of autophagic vacuoles by monodansyl cadaverine (MDC)

Fluorescent probe MDC is a selective marker for acidic vesicular organelles to evaluate autophagy. MCF7 cells grown on coverslips were treated with 5 μM 4-OHT for 48 h. Cells were then exposed to 50 μM MDC (Sigma-Aldrich) at 37 °C for 15 min in the dark for staining. After washing with PBS, cells were visualized under fluorescence microscope (Leica, Wetzlar, Germany).

### Plasmid construction and luciferase reporter assay

To generate luciferase constructs for evaluating miRNA activity, 3’ UTR of UCP2 gene was amplified using the primers whose sequences were 5’-gcGAGCTCgcctctcctgctgctgacc-3’and 5’-gcTCTAGAatggatgacaagtgggctagg-3’. PCR was performed with genomic DNA and digested by both Sac I and Xba I and cloned into a modified pGL3-control vector pISO provided by Dr. Zhihua Liu [26]. A pair of primer 5’-gcGAGCTCgcctctcctgatactgacctgatcacc-3’ and 5’-gcTCTAGAatggatgacaagtgggctaggctggg-3’ was used to generate mutation in the seed region binding sites. Wild type and mutant inserts were confirmed by sequencing.

For luciferase reporter assay, MCF7 cells seeded in a 96-well plate were co-transfected with firefly luciferase constructs (100 ng) and 40 nM miRNA mimics using lipofectamine 2000 reagent. Cells were also transfected with 10 ng of pRL-SV40 plasmid to monitor transfection efficiency. 36 h after transfection, firefly luciferase activity was evaluated by using a dual-luciferase reporter assay system (Promega Corporation). *Renilla* luciferase activity was evaluated to normalize firefly luciferase activity for each sample. Transfections were performed in triplicate and the experiments were repeated twice.

### Immunofluorescence staining analysis

MCF7 cells grown on cover-slips were fixed in 4 % paraformaldehyde for 10 min at room temperature. Cells were washed in PBS, blocked with 5 % bovine serum albumin (BSA) supplemented with 0.3 % Triton X-100 in PBS for 1 h. Cells were incubated with primary antibody (UCP2, Santa Cruz) in 1 % BSA at 4 °C overnight. After washing with PBS, cells were incubated with Rhodamine-labeled anti-goat secondary antibody (ZSGB-Bio, Beijing) in 1 % BSA for 1 h at room temperature. Cells were washed and cellular nuclei were stained with Hoechst 33342 (Sigma-Aldrich) for 10 min. Images were acquired under confocal microscope (TCS SP5, Leica).

### Statistical analysis

All data are presented as mean ± SD. Statistical differences were evaluated by analysis of variance (ANOVA) followed by Dunnett (multiple comparisons to the same control) post hoc tests. Values of *P* < 0.05 were considered as statistically significant.

## Results

### 4-OHT/FUL treatment induces autophagy in breast cancer cells

**Fig. 1:**
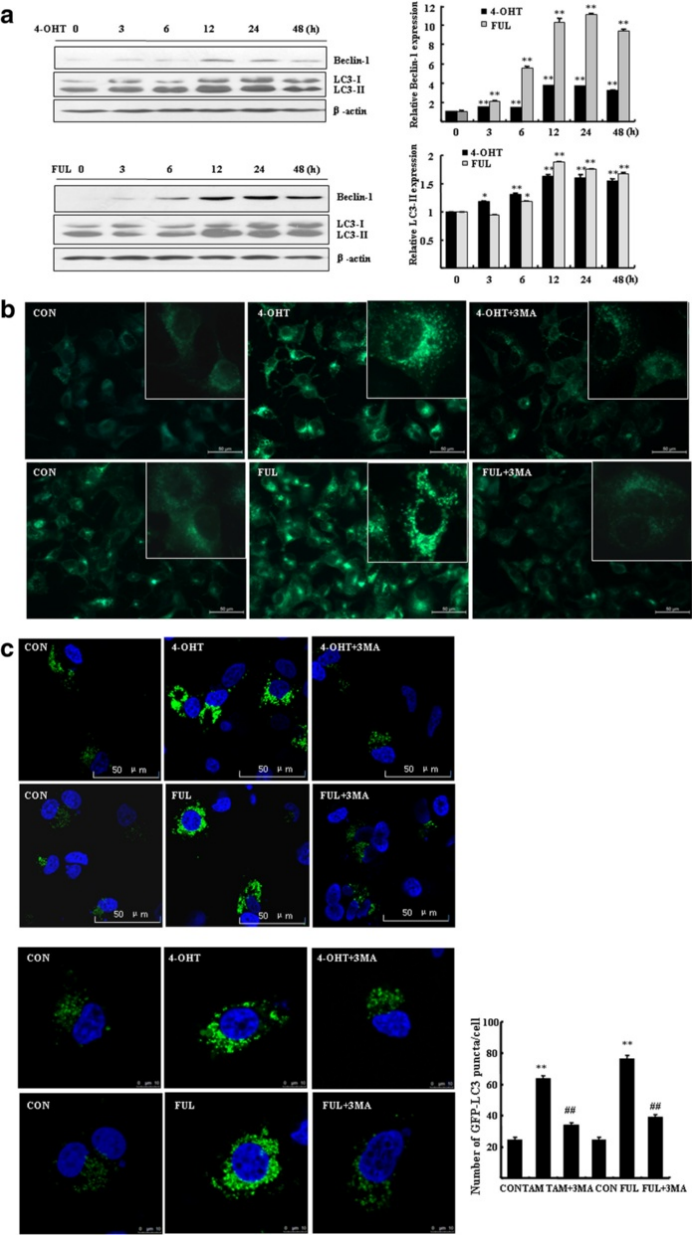
4-OHT or FUL induced autophagy in breast cancer cells. **a** MCF7 cells were exposed to 5 μM 4-OHT or 1 μM FUL for the indicated time. Western blotting was performed to determine the expressions of LC3 and Beclin-1. Bar graphs indicated relative levels of LC3-II and Beclin-1 normalized to β-actin. Data represent mean ± SD of three experiments. *P < 0.05, **P < 0.01 vs. vehicle control. **b** MCF7 cells seeded on coverslips were treated with 5 μM 4-OHT or 1 μM FUL for 48 h. After staining with MDC, cells were imaged under a fluorescence microscope (scale bar = 50 μm), with a magnified image showing punctate pattern. **c** MCF7 cells were transfected with GFP-LC3 for 24 h and then were treated with 5 μM 4-OHT or 1 μM FUL with or without 5 mM 3-MA for 48 h. Cells were imaged under a confocal microscopy, scale bar = 50 μm (*up*) or 10 μm (*bottom*). Numbers of GFP-LC3 puncta per cell were counted. **P < 0.01 vs. vehicle control, ^##^ *P* < 0.01 vs. TAM/FUL treatment, *n* = 10 cells. Data represent mean ± SD of three experiments

Human ER^+^ MCF7 cells were exposed to 5 μΜ 4-OHT, an active metabolite of TAM, or 1 μΜ FUL for different time and then the characteristics of autophagy was analyzed. Beclin-1 is one of markers that critically indicate the process of autophagosomic-lysosomal degradation of proteins activated in response to pathological disorders. 4-OHT and FUL treatment increased the expression of beclin-1 time-dependently. The increase of beclin-1 reached a peak at 12 h after exposure and remained at high levels up to 48 h (Fig. 1a). LC3-II, the cleaved and lipidated form of the microtubule associated protein light chain 3 (MAP1LC3), is the hallmark protein signifying the increase of autophagy. 4-OHT/FUL treatment led to elevation of LC3-II, which reached plateau during time from 12 to 48 h.

The 4-OHT/FUL-induced autophagy was also seen after staining with acidic vesicular organelles (AVOs). Figure 1b showed the morphological change of autophagy in the 4-OHT/FUL-treated cells. A significant increase of MDC fluorescent intensity was observed in the 4-OHT/FUL-treated cells, showing an increased fraction of cells with punctate staining distributed within cytoplasm or perinuclear regions. We added 3-MA, an autophagy inhibitor, to the 4-OHT/FUL-treated cells. The formation of autophagosomes was obviously inhibited (Fig. 1b).

MCF7 cells were transfected with GFP-LC3 plasmid and GFP-LC3-II puncta was analyzed in autophagic cells. The number of autophagosomes (GFP-LC3-II dots) was then counted in the 4-OHT/FUL-treated cells. 4-OHT/FUL treatment significantly increased GFP-LC3 puncta in MCF7 cells (Fig. 1c). However, in the presence of 3-MA, the formation of autophagosomes was inhibited (Fig. 1c, bottom, *P* < 0.01, vs. vehicle control).

### MiR-214 increases the 4-OHT/FUL-induced cell apoptosis

**Fig. 2:**
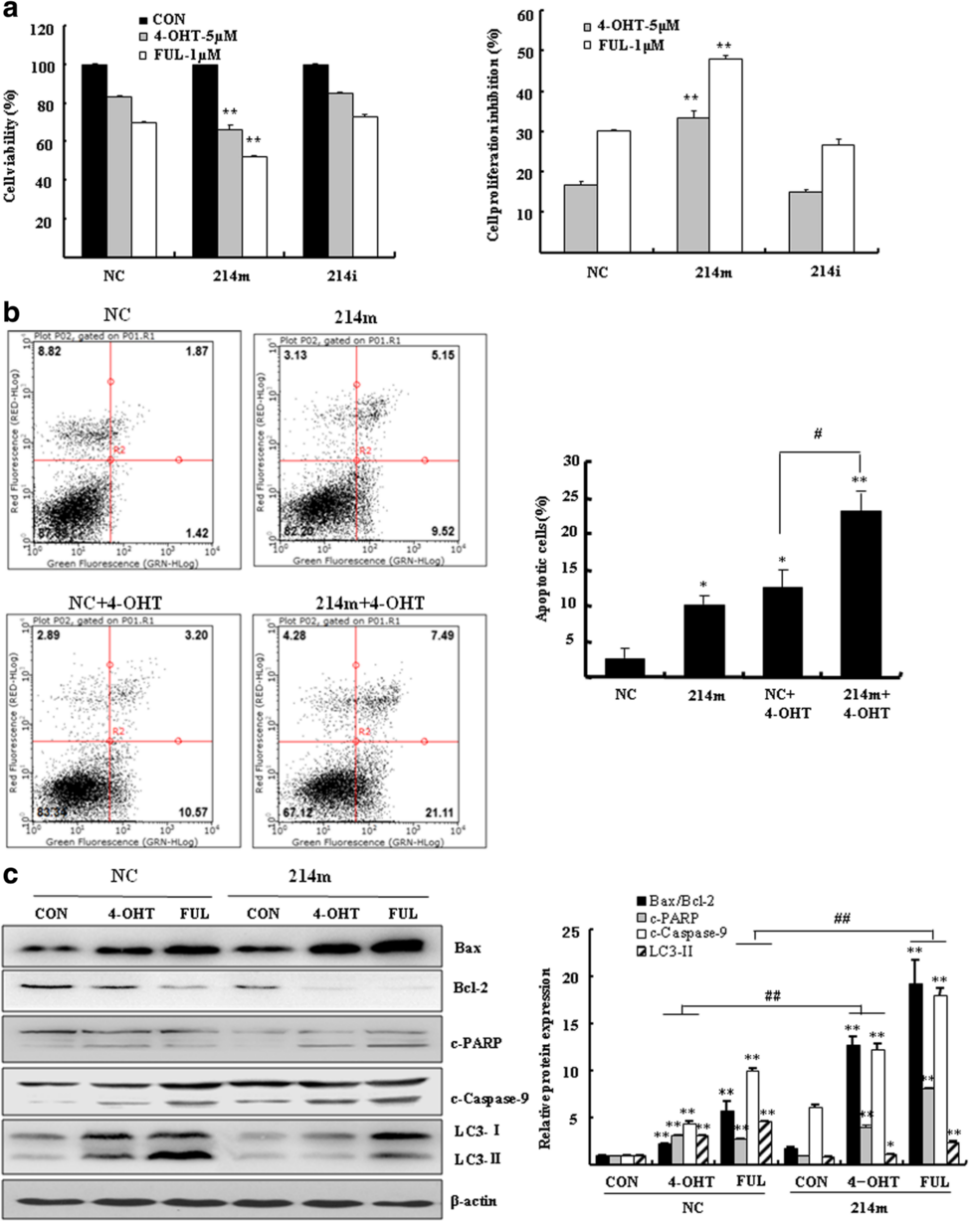
MiR-214 increased the TAM/FUL-induced apoptosis in breast cancer cells. **a** MCF7 cells were transfected with 100 nM miR-214 mimics (214 m) or inhibitors (214i) for 24 h. Cells were then treated with 5 μM 4-OHT or 1 μM FUL for 72 h. Cell viability was estimated by the MTT assay. **b** MCF7 cells were transfected with 100 nM miR-214 mimics (214 m) or negative control (NC). Cells were then treated with or without 5 μM 4-OHT for 48 h. Annexin V-PI double staining was performed by flow cytometry. Bar graphs indicated the percentage of apoptotic cells. Data represent mean ± SD of three experiments. *P < 0.05, **P < 0.01 vs. negative control (NC), ^#^ *P* < 0.05 vs. negative control treated with 4-OHT (NC + 4-OHT) (**c**) Western blotting was performed to analyze the expression of apoptotic proteins Bax, Bcl-2, c-PARP (cleaved PARP), cleaved-caspase-9 (c-capase-9) and autophagic protein LC3-II. Bar graphs indicated relative levels of Bax/Bcl-2, c-PARP, c-capase-9 and LC3-II normalized to β-actin. *P < 0.05, **P < 0.01 vs. vehicle control, ^##^ *P* < 0.01 vs. negative control (NC)

By binding to ER, the effect of 4-OHT/FUL mainly depends on the activity of apoptotic induction. In order to find miRNAs that sensitize the 4-OHT/FUL-induced apoptosis, cell proliferation and apoptosis were examined in the miRNAs-transfected MCF7 cells. MiR-214 was considered as an important mediator in the sensitization of the 4-OHT/FUL-induced apoptosis. The proliferation of MCF7 cells was inhibited by 16.7 ± 1.1 % by 4-OHT (Fig. 2a, *P* < 0.01 vs. vehicle control) and 30.0 ± 0.6 % by FUL (*P* < 0.01 vs. vehicle control). When MCF7 cells were transfected with miR-214 mimics, the percentage of inhibition by 4-OHT or FUL was significantly increased as compared to negative control cells (33.3 ± 2.1 % by 4-OHT, and 47.8 ± 1.1 % by FUL, respectively, *P* < 0.01). These results indicated that MCF7 cells transfected with miR-214 mimics exhibited higher sensitivity to 4-OHT/FUL than the cells transfected with negative control miRNA. However, miR-214 inhibitor did not significantly affect the viability of MCF7 cells treated with 4-OHT/FUL.

MiR-214 increased the effect of the 4-OHT-induced apoptosis. As shown in Fig. 2b, the percentage of apoptotic cells was 12.4 ± 2.6 % (*P* < 0.05 vs. negative control) in MCF7 cells exposed to 4-OHT. Cells transfected with miR-214 mimics demonstrated higher levels of apoptosis than negative control cells (*P* < 0.05). When these cells were exposed to 4-OHT, the percentage of apoptotic cells was significantly increased to 23.2 ± 2.9 % (*P* < 0.01 vs. negative control). There was a significant difference in the percentage of apoptotic cells between the miR-214-transfected cells and negative control cells in response to 4-OHT treatment (Fig. 2b, *P* < 0.05).

The effect of miR-214 mimics in improving the sensitization of breast cancer cells to the 4-OHT-induced apoptosis was further evaluated at the protein levels. Both 4-OHT and FUL significantly increased the ratio of Bax/Bcl-2, the levels of cleaved PARP and cleaved caspase-9 in MCF7 cells. Cells transfected with miR-214 mimics had higher levels of apoptotic proteins than negative control cells in response to 4-OHT or FUL exposure (Fig. 2c, *P* < 0.01 vs. vehicle control). Since autophagy plays a prosurvival role and contributes to drug resistance, it is likely that miR-214 mimics might enhance the sensitivity of breast cancer cells to the 4-OHT/FUL-induced apoptosis by the inhibition of autophagy. We analyzed the specific autophagy marker LC3 in the miR-214 mimics-transfected cells. As compared to control cells, the 4-OHT/FUL-induced cleavage of LC3-Ι into LC3-II was significantly attenuated in the miR-214 mimics-transfected cells (Fig. 2c, *P* < 0.01 vs. negative control).

### MiR-214 inhibits both basal and the 4-OHT/FUL-induced autophagy

**Fig. 3:**
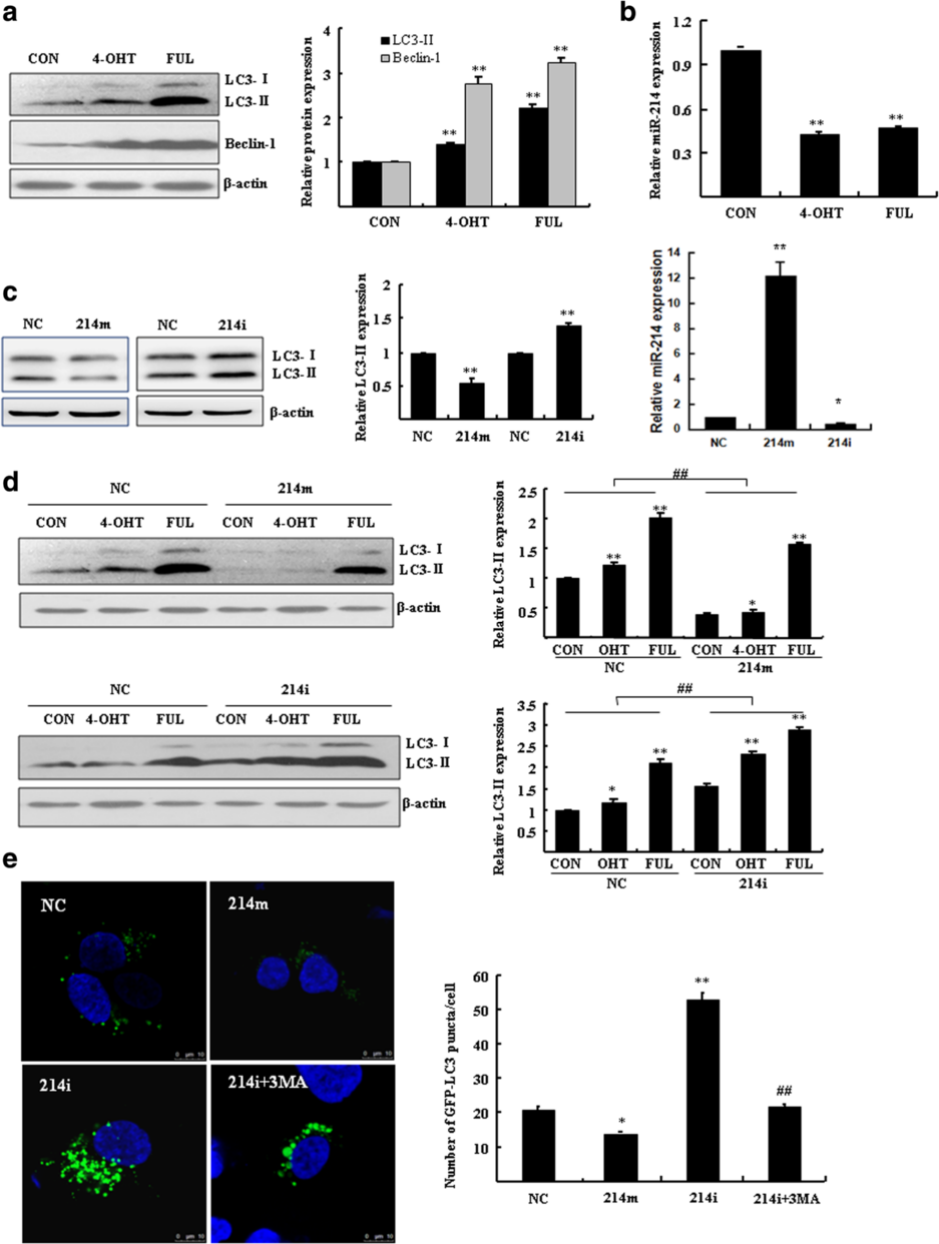
MiR-214 regulated autophagy at both basal and 4-OHT/FUL induced level. **a** MCF7 cells were treated with 5 μM 4-OHT or 1 μM FUL for 48 h. Western blotting was performed to analyze LC3 and Beclin-1. Bar graphs indicated relative levels of LC3-II and Beclin-1 normalized to β-actin. **P < 0.01 vs. vehicle control. **b** The expression of miR-214 was analyzed by RT-qPCR. **P < 0.01 vs. vehicle control. **c** and **d**) MCF7 cells were transfected with 100 nM miR-214 mimics (214 m) or inhibitors (214i) for 24 h, then were treated with or without 5 μM 4-OHT or 1 μM FUL for 48 h. Western blotting was performed to analyze LC3-II normalized to β-actin. RT-qPCR was performed to analyze the level of miR-214 (**c**). *P < 0.05, **P < 0.01 vs. vehicle control, ^##^ *P* < 0.01 vs. negative control (NC). **e** MCF7 cells were co-transfected with GFP-LC3 and 100 nM miR-214 mimics or inhibitors for 48 h in the presence or absence of 5 mM 3-MA. Cells were imaged under a confocal microscopy (scale bar = 10 μm). Numbers of GFP-LC3 puncta per cell were counted (*P < 0.05 **P < 0.01 vs. negative control, ^##^ *P* < 0.01 vs. transfection of miR-214 inhibitors (214i), *n* = 10). Data represent mean ± SD of three experiments

The induction of autophagy by 4-OHT/FUL was further evidenced by the elevated levels of cleaved LC3-II and beclin-1 (Fig. 3a). In fact, the level of miR-214 in these 4-OHT/FUL-treated cells was obviously reduced as compared with the vehicle control cells (Fig. 3b). These results implied that miR-214 might play important roles in the regulation of the 4-OHT/FUL-induced autophagy. Further evidence was found by Western blotting assay in MCF7 cells transfected with miR-214 mimics or inhibitors. As shown in Fig. 3c, the level of LC3-II was significantly reduced in MCF7 cells overexpressing miR-214, whereas miR-214 inhibitors stimulated the expression of cleaved LC3-II (Fig. 3c). These results indicated that miR-214 inhibited autophagy at the basal level. When cells were exposed to 4-OHT or FUL, miR-214 mimics and inhibitors had opposite effects on autophagy. As shown in Fig. 3d, in response to 4-OHT or FUL, the miR-214 mimics-transfected MCF7 cells demonstrated lower levels of LC3-II than negative control cells (Fig. 3d, above). These autophagic markers were dramatically augmented in the MCF7 cells transfected with miR-214 inhibitors (Fig. 3d, bottom). These results were also evidenced by analysis of autophagosomes under confocal microscopy. The formation of autophagosomes (GFP-LC3 dots) was significantly inhibited in the miR-214 overexpressing cells (Fig. 3e up). On the contrary, the number of GFP-LC3 dots in the MCF7 cells transfected with miR-214 inhibitors was obviously increased. The increase of GFP-LC3 dots was significantly reduced by 3-MA (Fig. 3e bottom, *P* < 0.01).

### UCP2 is a direct target of miR-214

**Fig. 4:**
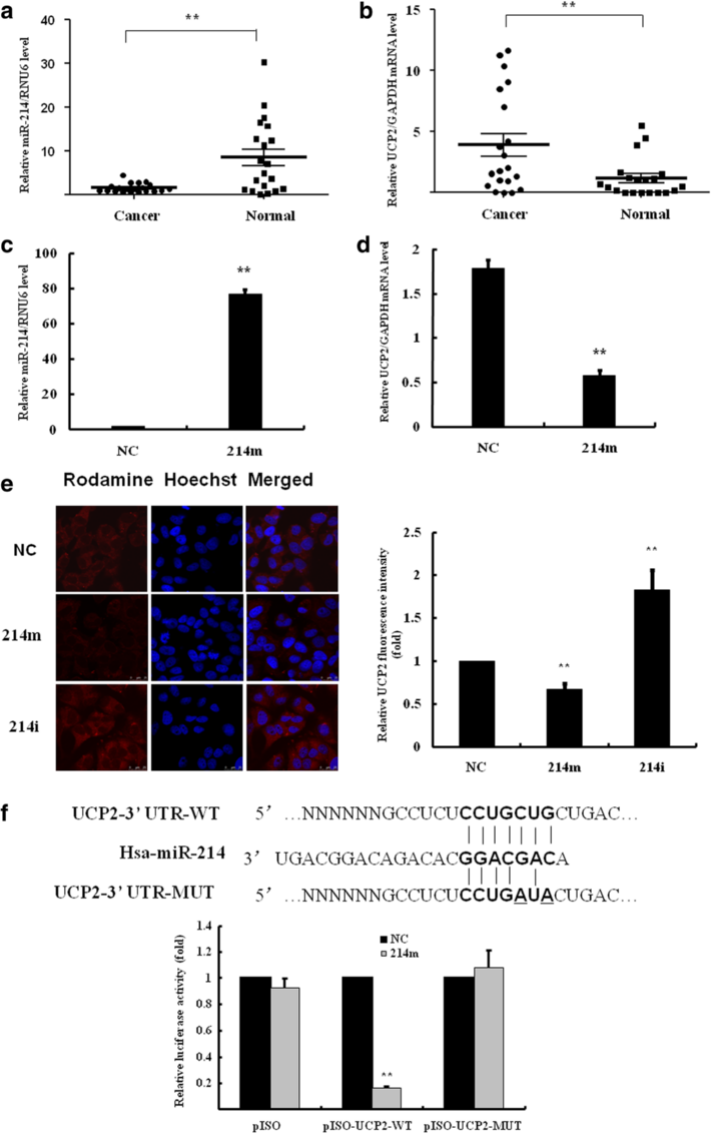
UCP2 was identified the target gene of miR-214. **a** RT-qPCR was performed to analyze the expression of miR-214 relative to RNU6 in 20 pairs of breast cancer tissues. **b** RT-qPCR was also performed to analyze the expression of UCP2 mRNA in breast cancer tissues. **c**, **d**) MCF7 cells were transfected with 100 nM miR-214 mimics, then RT-qPCR was performed to analyze the expression of miR-214 and UCP2 mRNA. **P < 0.01 vs. negative control (NC). **e** MCF7 cells were transfected with 100 nM miR-214 mimics or inhibitors for 48 h. The expression and location of the Rhodamine-labeled UCP2 were determined by the immunofluorescence assay. Hoechst 33342 was used to stain nuclei. Images were acquired by confocal microscopy. **f** MCF7 cells were transfected with luciferase constructs and miR-214 mimics. The comparison of luciferase activity of wild-type (WT) and mutant (MUT) UCP2 constructs was performed 36 h after transfection. Data was normalized to renilla activity. **P < 0.01 vs. negative control (NC)

Having established a link between miR-214 and autophagy, we further investigated the mechanism of miR-214 by identifying its downstream targets. We employed the TargetScan program to predict the potential target genes. To our interest, UCP2, a mitochondrial protein that has been implicated in the production of intracellular Reactive oxygen species (ROS), might be targeted by miR-214. To evidence this hypothesis, we analyzed the expression of miR-214 and UCP2 in human breast cancer tissue specimens. A potential correlation of miR-214 and UCP2 was observed in 20 pairs of human breast cancer tissues and matched normal tissues. Lower level of miR-214 was observed in most of cancer tissues (15/20, 75.0 %), while UCP2 mRNA was significantly upregulated (17/20, 85.0 %) as compared with the matched normal tissues (*P* = 0.0013 and *P* = 0.0022, respectively) (Fig. 4a, b). Further analysis showed that the level of miR-214 was negatively correlated with the elevation of UCP2 mRNA (Pearson *γ* = -0.49, *P* = 0.028).

To identify the target gene of miR-214, MCF-7 cells were transfected with miR-214 mimics or negative control. The level of UCP2 mRNA was significantly reduced in the miR-214 overexpressing cells (Fig. 4c, d). The inhibitory effect of miR-214 on the expression of UCP2 protein was also analyzed by immunofluorescence assay. Figure 4e showed the images of MCF7 cells with the Rhodamine-labeled UCP2 protein located in mitochondria. The fluorescence intensity was significantly reduced by 33.1 ± 7.6 % in the miR-214 mimics-transfected cells (*P* < 0.01 vs. negative control). In contrast, MCF7 cells transfected with miR-214 inhibitors produced a significant increase of 1.8 ± 0.3 fold in the fluorescence intensity of UCP2 as compared with negative control cells (*P* < 0.01 vs. negative control).

MiR-214 was found to downregulate UCP2 by targeting the site in 3’UTR of UCP2 gene determined by dual luciferase reporter assay (Fig. 4f). The binding of miR-214 to the target site in the 3’UTR of UCP2 was identified by cloning the wild-type (WT) and mutant UCP2 3’UTR behind the luciferase gene in pISO vector. Co-expression of UCP2-3’UTR constructs with miR-214 mimics in MCF7 cells resulted in an inhibition of the relative luciferase activity by 83.7 ± 1.1 % (*P* < 0.01) as compared with negative control miRNA mimics; whereas miR-214 mimics did not significantly inhibit luciferase activity in the cells expressing mutant UCP2-3’UTR. These results suggested that miR-214 might directly bind to the 3’UTR of UCP2 and modulate UCP2 expression.

### Overexpression of UCP2 contributes to endocrine resistance to 4-OHT

**Fig. 5:**
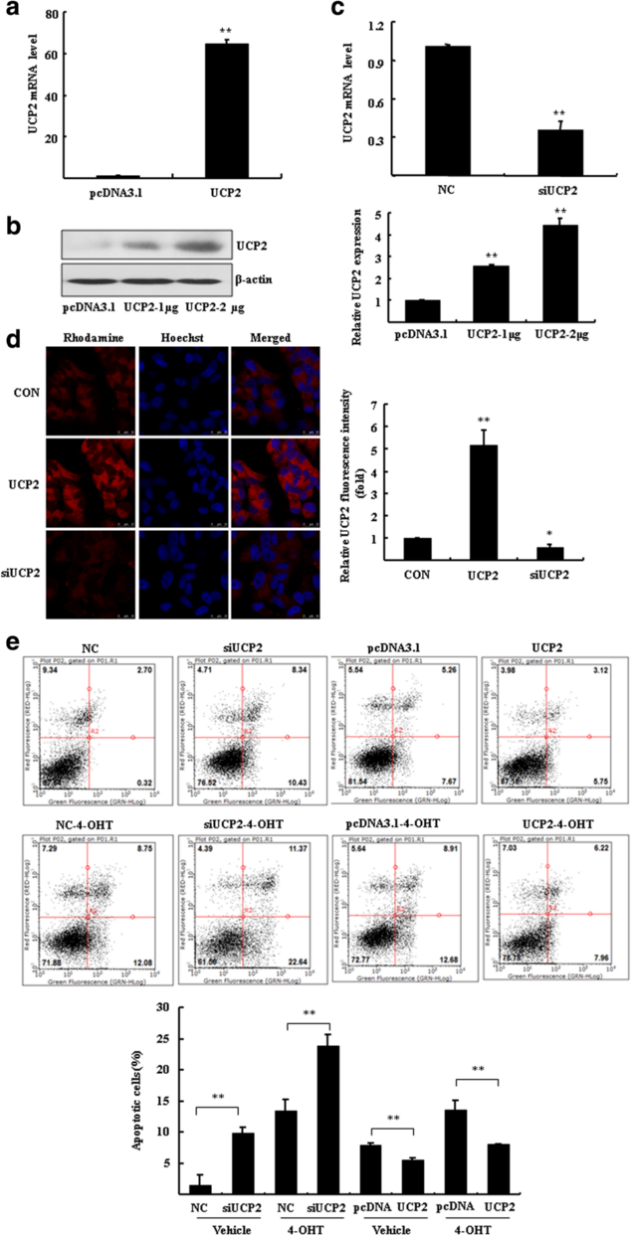
UCP2 modulated the 4-OHT/FUL-induced apoptosis in breast cancer cells. **a**-**c** MCF7 cells were transfected with UCP2 plasmid and empty vector pcDNA3.1 or 200 nM siRNA of UCP2 for 48 h. RT-qPCR and Western blotting were performed to determine the expression of UCP2. The ratio UCP2/β-actin is quantified (**P < 0.01 vs. empty vector control). **d** The immunofluorescence assay was performed to analyze the expression and location of UCP2 in MCF7 cells. **e** Annexin V-PI staining assay was performed to determine apoptosis in MCF7 cells. Bar graphs indicated the percentage of apoptotic cells. **P < 0.01 vs. negative control (NC) or empty vector control (pcDNA3.1)

Based on the observation of relevance between UCP2 and miR-214, we hypothesized that miR-214 might increase the 4-OHT-induced apoptosis through inhibition of UCP2. We explored the role of UCP2 in affecting the 4-OHT-induced apoptosis in breast cancer cells. UCP2 expression plasmids were constructed and then transfected into MCF7 cells. The expression of UCP2 was analyzed by RT-qPCR and Western blotting (Fig. 5a, b). RNA interference assay was performed to knockdown UCP2. Results showed that the level of UCP2 mRNA was significantly reduced by 65.1 ± 6.2 % (Fig. 5c, *P* < 0.01). Overexpression and knockdown of UCP2 by transfection of UCP2 expression plasmid and siRNA were analyzed by immunofluorescence staining assay (Fig. 5d). Transfection of UCP2 expression plasmids or UCP2 siRNA led to a pronounced increase or decrease of Rhodamine-labeled fluorescence intensity as compared with control cells (*P* < 0.05).

Subsequently, the apoptotic cells were determined by Annexin V-Propidium Iodide (PI) staining by flow cytometry. As shown in Fig. 5e, UCP2 siRNA led to a significant increase of apoptosis in the presence or absence of 4-OHT as compared to negative control (*P* < 0.01). In contrast, UCP2 overexpression attenuated both endogenous and the 4-OHT-induced apoptosis compared with empty vector control (*P* < 0.01). These results indicated that downregulation of UCP2 increased the sensitivity of breast cancer cells to 4-OHT, whereas overexpression of UCP2 contributed to acquired endocrine resistance to 4-OHT.

### PI3K-Akt-mTOR pathway might be involved in the induction of autophagy by overexpression of UCP2

**Fig. 6:**
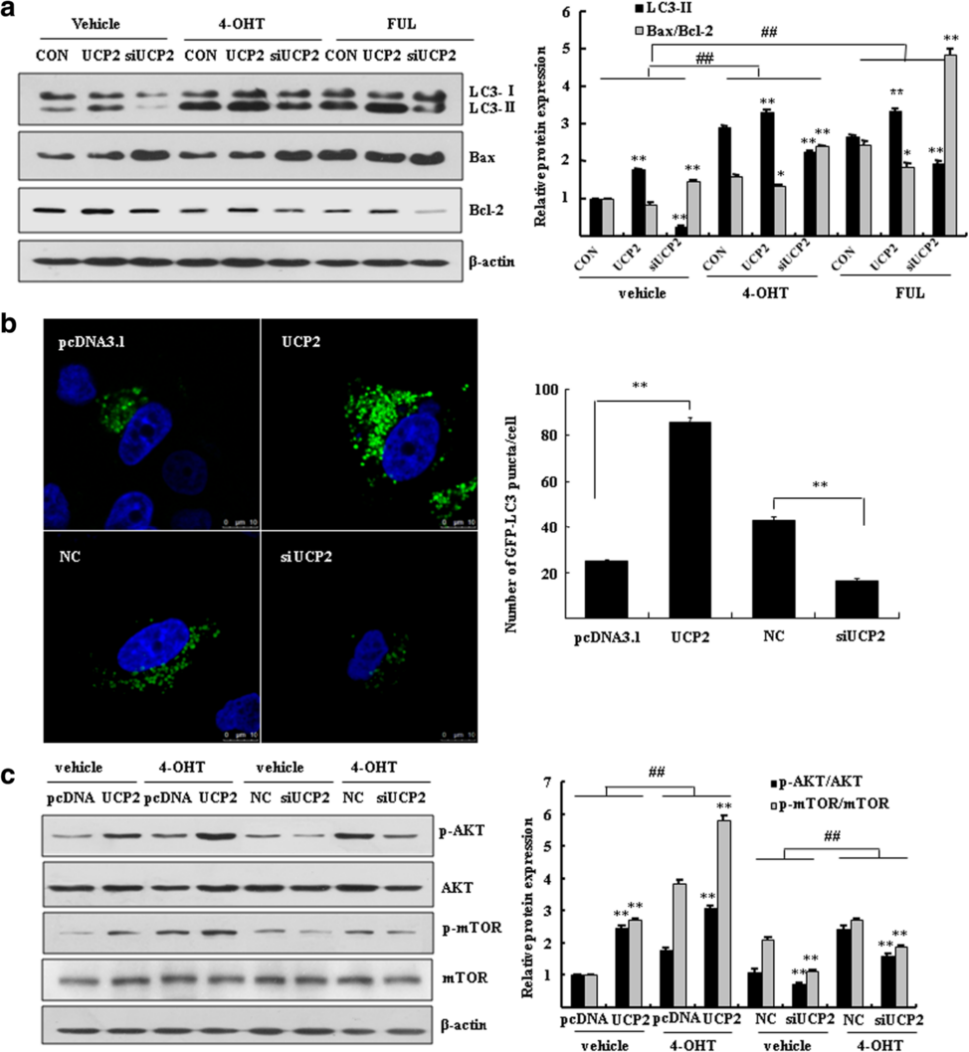
UCP2 modulated autophagy through activation of the PI3K-Akt-mTOR pathway. **a** MCF7/LCC9 cells were transfected with UCP2 plasmid or siRNA of UCP2 for 48 h. Western blotting was performed to analyze the expression of cleaved LC3 and the ratio of Bax/Bcl-2. Bar graphs indicated the relative levels of LC3-II and ratio of Bax/Bcl-2 normalized to β-actin. *P < 0.05, **P < 0.01 vs. control. ^##^ *P* < 0.01 vs. vehicle control. **b** MCF7 cells were cotransfected with GFP-LC3 together with UCP2 plasmid or 200 nM siRNA of UCP2 for 48 h. Cells were imaged under a confocal microscopy (scale bar = 10 μm). Numbers of GFP-LC3 puncta per cell were counted (**P < 0.01 vs. empty vector control or negative control, *n* = 10). **c** Western blotting was performed to analyze p-Akt, Akt, p-mTOR, mTOR in MCF7/LCC9 cells. β-actin was served as loading control. Bar graphs indicated the relative levels of p-Akt/Akt and p-mTOR/mTOR normalized to β-actin. ^**^ *P* < 0.01 vs. empty vector control or negative control, ^##^ *P* < 0.01 vs. vehicle control

We investigated the role of UCP2 in the autophagy that confers endocrine resistance. TAM and FUL cross-resistant MCF7/LCC9 cells were employed in the assays. MCF7/LCC9 cells were transfected either with UCP2 plasmid or with siRNA and then exposed to 4-OHT or FUL. High level of cleaved LC3-II was observed in the UCP2 overexpressing cells, whereas low level of active form of LC3-II was detected in the UCP2 knockdown cells. These results indicated that overexpression of UCP2 induced cell autophagy in the 4-OHT/FUL-treated MCF7/LCC9 cells. Consistently, the ratio of Bax//Bcl-2 was significantly elevated in the UCP2-knockdown cells (Fig. 6a), indicating the increase of apoptosis. The induction of autophagy by UCP2 was further determined by the observation of punctate GFP-LC3 distribution in breast cancer cells. Overexpression of UCP2 induced prominent autophagy as indicated by enhanced GFP-LC3 dots. In contrast, knockdown of UCP2 inhibited the formation of autophagesomes as shown by punctate GFP-LC3, accounting for 38.3 ± 8.8 % of negative control (Fig. 6b).

To investigate the signaling pathway of autophagy regulated by UCP2, MCF7/LCC9 cells were overexpressed or knockdown UCP2 and then exposed to 4-OHT. MCF7/LCC9 cells overexpressing UCP2 showed activation of Akt and mTOR in response to 4-OHT, whereas the expressions of phospho-Akt and phospho-mTOR were significantly decreased in the UCP2-knockdown cells (Fig. 6c). Significant changes of non-phosphorylated Akt and mTOR were not observed between the UCP2-overexpressing and UCP2-knockdown cells. These results indicated that the activation of the PI3K-Akt-mTOR signaling pathway might be involved in the induction of autophagy by overexpression of UCP2 in breast cancer cells.

## Discussion

The development of resistance to endocrine therapy is the biggest challenge for treatment of breast cancers with TAM or FUL. Endocrine resistance to TAM or FUL was found to link with multiple reasons, such as loss of ER, apoptosis resistance and altered expression of miRNAs [27]. Recently, studies have been focused on the aberrant regulation of autophagy in breast cancers. TAM/FUL treatment led to apoptosis as well as autophagy in the ER^+^ breast cancers [14]. Autophagy as a cytoprotective adaptive response usually occurs in the presence of apoptosis inductors. Molecular analysis indicated that Beclin-1, the key player in autophagy, can bind to Bcl-2 or another anti- apoptotic molecule, Bcl-xL. Beclin-1 is therefore activates the initial stages of autophagy when it is dissociated from Bcl-2 [28, 29]. In this study, miR-214 increased the sensitivity of breast cancer cells to the TAM/FUL-induced apoptosis. Importantly, miR-214 increased the TAM/FUL-induced apoptosis while inhibited autophagy. Further study showed that miR-214 might exert its inhibitory effect on autophagy by targeting UCP2. This result was confirmed by our observation in human breast cancer tissue specimens that the levels of miR-214 were negatively correlated with UCP2. Overexpression of UCP2 contributed to endocrine resistance against TAM/FUL. We thus concluded that miR-214 inhibited the TAM/FUL induced autophagy through suppression of UCP2. MiR-214 might be developed as a novel strategy for overcoming endocrine resistance in breast cancers. These results provided new insights into the mechanism of endocrine resistance by modulation of autophagy.

Autophagy is an evolutionarily conserved catabolic process for degradation of cytoplasmic components including defective organelles and proteins. Physiologically, autophagy is implicated in cellular homeostasis by recycling of nutrients whereas defect of autophagy is associated with cancers [30, 31]. Autophagy can act as a pro-survival mechanism or as an alternative cell death pathway to apoptosis [28]. Despite duality of autophagy, accumulated evidence revealed an important role of autophagy in the development of endocrine resistance in the ER^+^ breast cancers [18]. Recent studies showed that the inhibition of autophagy might increase the 4-OHT-induced cytotoxicity whereas increase of autophagy is associated with endocrine resistance to 4-OHT [32]. In breast cancer cells, inhibition of autophagy by 3-MA or beclin-1 siRNA potentiated the resensitization of previously antiestrogen resistant breast cancer cells [6, 14], suggesting a pro-survival role of autophagy in anti-estrogen therapy.

MiRNAs are involved in the regulation of many autophagic processes, including autophagy induction, vesicle nucleation and vesicle elongation and fusion, which provides a potential for combining miRNA-based manipulation of autophagy with therapy resistance in cancers [33]. Inhibition of beclin 1-mediated autophagy by miR-30d mimic sensitized anaplastic thyroid carcinoma cells to cisplatin [34]. MiR-101 suppressed the tamoxifen-induced autophagy and increased the sensitivity of breast cancer cells to tamoxifen [19]. In this study, miR-214 was found to sensitize breast cancer cells to 4-OHT or FUL by inhibition of autophagy.

Accumulating studies have shed light on the potential of miR-214 as the therapeutic strategy for cancers. MiR-214 was found downregulated in breast cancers [22]. MiR-214 might play pleiotropic and pivotal roles in resistance or sensitivity in various cancers. On one hand, high level of miR-214 enhanced the stemness and chemoresistance in ovarian cancers by targeting p53/Nanog [35]. MiR-214 might be responsible for chemoresistance to cisplatin or gefitinib in ovarian cancer and non small cell lung cancer [36, 37]. On the other hand, miR-214 enhanced the cisplatin-induced cytotoxicity through down-regulation of Bcl2l2 in cervical cancer cells [38]. In this study, miR-214 was found downregulated in human breast cancer tissues and decreased in response to 4-OHT/FUL. Our results showed that reduction of miR-214 was associated with elevated autophagy and endocrine resistance, while overexpression of miR-214 represses both basal and the 4-OHT/FUL-induced autophagy. Further study showed that miR-214 might reduce autophagy by directly targeting UCP2.

UCP2 is a member of the family of uncoupling proteins located in inner mitochondrial membrane. UCP2 is involved in uncoupling oxidative phosphorylation and facilitating energy dissipation as heat [39]. Overexpression of UCP2 conferred drug resistance to chemotherapy and a higher survival through downregulation of ROS [40, 41]. UCP2 was thus recognized as a negative regulator of mitochondrial ROS in many type of cancers [42]. Overexpression of UCP2 decreased intracellular ROS and attenuated apoptosis in HepG2 hepatoma cells induced by various challenges [43]. Overexpression of UCP2 protected cancer cells from the gemcitabine-induced apoptosis through decrease of mitochondrial superoxide [44]. Inhibition of UCP2 enhanced the cisplatin-induced cytotoxicity in colon cancer cells. UCP2 has been identified as a potential target for overcoming cancer chemoresistance [45]. In this study, breast cancer cells with knockdown of UCP2 showed higher sensitivity to 4-OHT than negative control cells in induction of apoptosis, whereas overexpression of UCP2 attenuated the activity of 4-OHT. Further study showed that UCP2 might play a crucial role in the induction of autophagy that confers the endocrine resistance in the TAM and FUL cross-resistant MCF7/LCC9 cells.

**Fig. 7:**
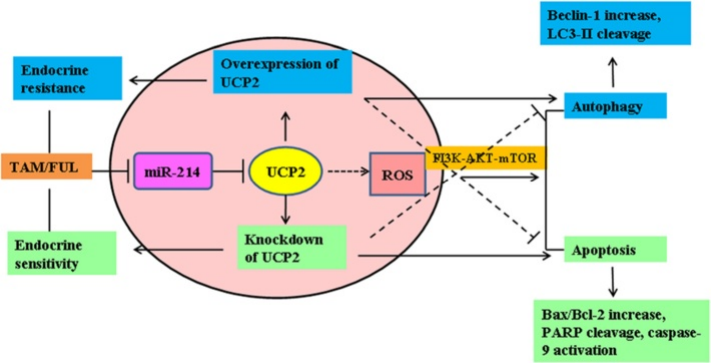
A summary of the relationships between miR-214, UCP2, beclin-1, LC3-II, Bax, Caspase-9, PARP, and PI3K-Akt-mTOR after TAM/FUL treatment. TAM/FUL treatment results in autophagy as well as apoptosis. MiR-214 increased the sensitivity of breast cancer cells to the 4-OHT/FUL-induced apoptosis through inhibition of autophagy. UCP2 was identified to be a direct target of miR-214. Overexpression of UCP2 inhibited TAM/FUL-induced cell apotosis by increasing autophagy possibly through activation of the PI3K-Akt-mTOR signaling pathway, thereby contributing to endocrine resistance

Recently, the PI3K/AKT/mTOR signaling pathway, which transmits signals from cell membrane into nucleus and activates multiple oncogenic programs, has been reported to play an important role in the regulation of autophagy in breast cancer cells. This signaling pathway is hyperactive in more than 70 % of breast cancers. It has been revealed that the PI3K/AKT/mTOR signaling modulates the function of estrogen receptor. Activation of the PI3K-AKT-mTOR signaling is relevant to the escape of cancer cells from endocrine therapy by inhibiting the proapoptotic proteins and therefore contributes to endocrine resistance [46, 47]. Hence, the protein kinases located along this signaling pathway represent very promising drug targets for breast cancer therapy. In this study, UCP2 was found overexpressed in breast cancer tissue specimens. Overexpression of UCP2 induced autophagy and endocrine resistance through phosphorylation of Akt and mTOR. These results suggested that the activation of the PI3K-Akt-mTOR signaling pathway might contribute to the UCP2-mediated autophagy in breast cancers. Nonetheless, there were some contradictory reports that inhibition of the PI3K-Akt-mTOR signaling pathway induced autophagy in cancer cells [12]. It has been well established that ROS modulated the autophagy process in cancer progression and chemotherapy [48, 49]. It is hypothesized that regulation of UCP2, a critical mitochondrial protein involved in ROS production, might play a key role in coordinating autophagy and endocrine resistance through the PI3K-Akt-mTOR signaling pathway [50]. Based on currently available information, we have proposed a schematic model between miR-214, UCP2, beclin-1, LC3-I/II, Bax, Caspase-9, PARP, and Akt/mTOR in the regulation of autophagy and apoptosis after TAM/FUL treatment (Fig. 7). Further studies are underway to elucidate the mechanism of UCP2-mediated autophagy in breast cancers.

## Conclusion

In summary, TAM and FUL treatment induced apoptosis as well as autophagy in the ER^+^ breast cancer cells. Autophagy is a major cause of resistance to TAM and FUL. MiR-214 increased the sensitivity of breast cancers to TAM and FUL through inhibition of autophagy by targeting UCP2. MiR-214 could be developed as a novel potential therapeutic strategy for overcoming endocrine resistance in the ER^+^ breast cancers.

## Abbreviations

3-MA: 3-methyladenine
4-OHT: 4- hydroxytamoxifen
AVOs: acidic vesicular organelles
ER: estrogen receptor
FUL: fulvestrant
HER2: human epidermal growth receptor 2
MDC: monodansyl cadaverine
miRNAs: microRNAs
PR: progesterone receptor
TAM: tamoxifen
UCP2: uncoupling protein 2
